# If host is refractory, insistent parasite goes berserk: Trypanosomatid *Blastocrithidia raabei* in the dock bug *Coreus marginatus*

**DOI:** 10.1371/journal.pone.0227832

**Published:** 2020-01-16

**Authors:** Alexander O. Frolov, Marina N. Malysheva, Anna I. Ganyukova, Viktoria V. Spodareva, Jana Králová, Vyacheslav Yurchenko, Alexei Y. Kostygov

**Affiliations:** 1 Zoological Institute of the Russian Academy of Sciences, St. Petersburg, Russia; 2 Life Science Research Centre, Faculty of Science, University of Ostrava, Ostrava, Czech Republic; 3 Martsinovsky Institute of Medical Parasitology, Tropical and Vector Borne Diseases, Sechenov University, Moscow, Russia; Universidade Federal do Rio de Janeiro, BRAZIL

## Abstract

Here we characterized the development of the trypanosomatid *Blastocrithidia raabei* in the dock bug *Coreus marginatus* using light and electron microscopy. This parasite has been previously reported to occur in the host hemolymph, which is rather typical for dixenous trypanosomatids transmitted to a plant or vertebrate with insect's saliva. In addition, *C*. *marginatus* has an unusual organization of the intestine, which makes it refractory to microbial infections: two impassable segments isolate the anterior midgut portion responsible for digestion and absorption from the posterior one containing symbiotic bacteria. Our results refuted the possibility of hemolymph infection, but revealed that the refractory nature of the host provokes very aggressive behavior of the parasite and makes its life cycle more complex, reminiscent of that in some dixenous trypanosomatids. In the pre-barrier midgut portion, the epimastigotes of *B*. *raabei* attach to the epithelium and multiply similarly to regular insect trypanosomatids. However, when facing the impassable constricted region, the parasites rampage and either fiercely break through the isolating segments or attack the intestinal epithelium in front of the barrier. The cells of the latter group pass to the basal lamina and accumulate there, causing degradation of the epitheliocytes and thus helping the epimastigotes of the former group to advance posteriorly. In the symbiont-containing post-barrier midgut segment, the parasites either attach to bacterial cells and produce cyst-like amastigotes (CLAs) or infect enterocytes. In the rectum, all epimastigotes attach either to the cuticular lining or to each other and form CLAs. We argue that in addition to the specialized life cycle *B*. *raabei* possesses functional cell enhancements important either for the successful passage through the intestinal barriers (enlarged rostrum and well-developed Golgi complex) or as food reserves (vacuoles in the posterior end).

## Introduction

The flagellates of the family Trypanosomatidae are globally distributed parasites inhabiting a very wide range of hosts: leeches, insects, vertebrates as well as plants and even ciliates [1, 2]. The research interest in this group is greatly stimulated because some of its members, belonging to genera *Trypanosoma* and *Leishmania*, cause severe diseases of humans and domestic animals (sleeping sickness, Chagas disease, nagana, surra, kala-azar, canine leishmaniasis, etc.) [3, 4]. Although insect-restricted (monoxenous) trypanosomatids do not have such a strong impact, studying them is as important because these species serve as models for the abovementioned pathogens and because of many peculiarities, inherent to the whole family: polycistronic transcription and *trans*-splicing of nuclear protein-encoding genes, complex organization of kinetoplast DNA, RNA editing, etc. [5]. In the last few years monoxenous species started attracting even more attention owing to the ability of some flagellates to survive in humans [6–11], negative effect on economically important insects [12, 13] and symbiotic relationships with intracellular bacteria [14–18]. The study of monoxenous trypanosomatids sheds light on the evolution of the whole family and dixenous life cycles (with two unrelated hosts, of which one is a vector) in particular [19–23]. However, many functional and genomic studies of these parasites lack the data on their development in the insect host. Because of this, the meaning of many observed features and phenomena remains unexplained.

One of the most interesting groups of monoxenous trypanosomatids is the genus *Blastocrithidia*. *Blastocrithidia* spp. have been recently demonstrated to possess a non-canonical genetic code with all threes stop codons encoding amino acids [24, 25]. In order to better understand the reasons of this and other peculiarities it is imperative to have data on parasites' development in their hosts. However, as for the majority of monoxenous trypanosomatids, the life cycles of *Blastocrithidia* spp. are largely unknown [26]. There are only two exceptions: *B*. *triatomae* from the predatory triatomine bugs and *B*. *papi* from the omnivorous firebug *Pyrrhocoris apterus* [27–30].

*Blastocrithidia* spp. is likely the most aggressive group among monoxenous trypanosomatids and, therefore, were considered as potential tools for biological control [31]. For instance, *B*. *triatomae* infection causes high mortality in nymphs of the kissing bug, *Triatoma infestans* [32]. This parasite is able to destroy the midgut epithelium and induce pathological changes in Malpighian tubules [33, 34]. The development of *B*. *papi* in the Malpighian tubules of the firebug *Pyrrhocoris apterus* leads to their obstruction and reduction of microvilli because of intensive proliferation and attachment of epimastigotes, respectively [30].

Here we address biology of one more species of this genus, *Blastocrithidia raabei*, which was originally described from the intestine and hemolymph of the dock bug *Coreus marginatus* L. (Heteroptera: Coreidae) in Poland [35] and later from the same host in Kazakhstan, Tajikistan, and Armenia as the subspecies *B*. *raabei rostrata* [36]. Our interest in this parasite was stimulated by the Lipa's suggestion that it may occur in the insect's hemolymph. Such a localization is typical for some dixenous trypanosomatids (transmitted to a plant or a vertebrate with insect's saliva), but is very rare for monoxenous species [37]. In addition, the bug *Coreus marginatus* is strictly phytophagous. Its midgut has two specialized impassable parts (the "constricted region" and M4B) isolating anterior segments, used for digestion and absorption, from the posterior one, which bears bacterial symbionts [38]. Although many monoxenous trypanosomatids are known to inhabit various related plant-sucking stinkbugs [1], the host-parasite relationships in such parasitic systems have not been investigated. Here we studied relationships between *B*. *raabei* and *C*. *marginatus* in natural infections. Our results demonstrate that although the parasite does not inhabit hemolymph, in many respects its outstandingly aggressive behavior is similar to that of some dixenous trypanosomatids. The barriers that the host sets on the way of this species force the flagellate to "go berserk".

## Material and methods

### Collection and dissection of insects

**Fig 1:**
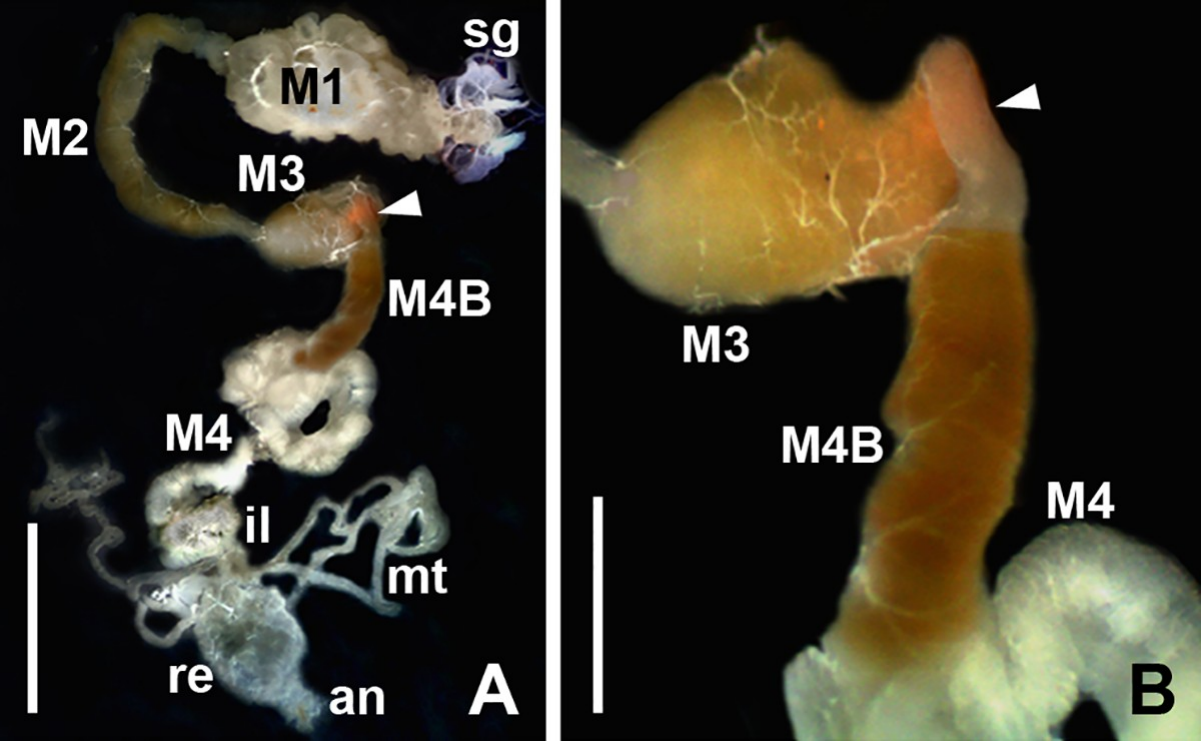
Isolated digestive system of *Coreus marginatus* (*ex vivo*, reflected light). **A.** General view. **B.** M3 –M4 segments of the midgut at higher magnification. **M1** –**M4** the corresponding midgut segments; **an**–anus; **il**–ileum; **mt**–Malpighian tubules; **re**–rectum; **sg**–salivary glands. Arrowhead–constricted region. Scale bars: 3 mm (**A**); 0.7 mm (**B**).

The dock bugs *Coreus marginatus* were collected from May to September in 2016–2019 from the vegetative and reproductive parts of the Russian dock (*Rumex confertus*), the bitter dock (*R*. *obtusifolius*), and the false rhubarb (*Rheum rhaponticum*). Insects were collected in the North-West of the European part of Russia (Leningrad Oblast, village Apraksin, 59°46' N, 31°12' E; Pskov Oblast village Lyady, 58°35' N, 28°55' E; and Novgorod Oblast, village Oksochi, 58°39' N, 32°47' E) and in the southern part of Western Siberia (Kurgan Oblast, village Zaozerny, 55°28' N, 65°16' E). In total, 141 imagines were examined. The bugs were immobilized with chloroform vapors and their hemolymph was sampled and analyzed as described previously [39]. The insects were dissected in a saline solution; the salivary glands and intestine were prepared and analyzed as described previously [29, 30]. All midgut segments (M1-M4 and M4B), ileum, Malpighian tubules and rectum were examined separately (Fig 1A and 1B). The trypanosomatid-containing material was used for preparation of dry smears, establishing cultures, DNA isolation, and electron microscopy.

In addition, we analyzed one isolate collected in 2010 in Southern Ural (Orenburg Oblast, near village Churaevo, 51°38'N, 57°31'E) preserved in the collection of the Zoological Institute of the Russian Academy of Sciences as a Giemsa-stained smear and total genomic DNA isolated from the whole midgut.

The dock bugs' collection did not require specific permissions, since they were sampled in the localities of public access, and *C*. *marginatus* is not an endangered or protected species.

### Cultivation and cryopreservation of trypanosomatids

Six axenic cultures of *B*. *raabei* were established in TC-100 Insect medium (Sigma-Aldrich, St. Louis, USA) supplemented with 10% Fetal Bovine Serum (BioloT, St. Petersburg, Russia) and passaged monthly at 20°C. Antibiotics (500 μg/ml of Streptomycin and 500 Units/ml of Penicillin) were added to the medium only at the first passage. Purification of cultures from fungal contamination was achieved using the previously described device consisting of two glass tubes with a V-shaped cannular connector [40]. In total, 6 cultures were cryopreserved and stored at -86° C in the growth medium, supplemented with 10% DMSO (Sigma-Aldrich).

### Microscopy

The smears prepared from the contents of the infected organs were air-dried, fixed with 96% ethanol for 30 minutes, and stained with Giemsa or 4’,6-diamidino-2-phenylindole (DAPI) as described previously [41].

In this work we also used Giemsa-stained type smears of *Blastocrithidia raabei rostrata* prepared by Podlipaev in 1981 from the total abdominal contents of *Coreus marginatus* collected in Kazakhstan (village Zhabagly, 42° 26' N, 70° 29' E) and preserved in the collection of the Zoological Institute of Russian Academy of Sciences (# SP200–210).

Digital photos were taken using DM 2500 microscope (Leica Microsystems GmbH, Wetzlar, Germany) equipped with UCMOS14000KPA 14-Mpx camera (Toup Tek, Hangzhou, China) at ×1,000 magnification. All cell measurements (n = 25) and statistical analysis were performed in UTHSCSA Image Tool for Windows v. 3.0. For transmission and scanning electron microscopy the samples were fixed and processed as described previously [30].

### DNA isolation, amplification, cloning, and sequencing

Total genomic DNA was extracted from the field samples with DNeasy Blood & Tissue Kit (Qiagen, Hilden, Germany) according to the manufacturer’s instructions. SSU rRNA gene was amplified using S762 and S763 primers [42] as described previously [43] and sequenced directly using the strategy reported elsewhere [44] at Macrogen Europe (Amsterdam, Netherlands). Primers M167 and M168 [45] were used to amplify the 5S / SL RNA repeat region. The resulting amplicons were cloned into pCR2.1 (Invitrogen, Carlsbad, USA) and sequenced using standard vector primers. The 16S rRNA gene of the bacterial symbionts, inhabiting the M4 midgut segment of dock bugs, was amplified with the universal eubacterial primers as described previously [46]. The GenBank accession numbers for the new sequences determined in this work are MN366346-MN366353 (18S rRNA gene), MN380289-MN380296 (5S / SL RNA repeat region) and MN365899-MN365900 (bacterial 16S rRNA gene).

### Phylogenetic analyses

#### 18S rRNA gene

Twenty eight 18S rRNA gene sequences of *Blastocrithidia* spp. and the closely related *"jaculum"* lineage [47, 48], used as an outgroup, were aligned in MAFFT v. 7.427 with the E-INS-i method [49]. Gaps present in over 50% sequences were removed by Gap Strip/Squeeze V. 2.1.0 (https://www.hiv.lanl.gov/content/sequence/GAPSTREEZE/gap.html).

Maximum likelihood analysis was performed in IQTREE v. 1.6.10 [50] under the TIM3e + I + G4 model as selected by the built-in ModelFinder [51]. Statistical support of bipartitions was estimated using the "standard" bootstrap test with 1,000 replicates. MrBayes 3.2.7 was used for Bayesian inference [52] under the GTR + I + G model (4 gamma categories) with 5 million generations and sampling every 1,000^th^ of them, whereas other parameters were set as default.

#### 5S / SL RNA gene region

All 5S / SL RNA gene region sequences of *Blastocrithidia* available in the Genbank along with those obtained in this work were aligned with MAFFT as above. The resulting alignment was manually refined in Bioedit [53]. The sequences were clustered in MEGA X software [54] using neighbor-joining method under K2P model with pairwise gap deletion and 1,000 bootstrap replicates.

## Results

Out of 141 dissected *Coreus marginatus* individuals, epimastigotes were detected in the intestine of 53 (~ 37.5%) imagines. Four individuals (~ 3%) had coinfection of *B*. *raabei* and *Phytomonas lipae* [55]. The prevalence of *B*. *raabei* varied between different regions and amounted to 52.0%, 46.3%, 32.4% and 30.8% in Leningrad, Pskov, Novgorod and Kurgan Oblasts, respectively.

### Parasite's morphology in the intestine

#### Light microscopy

Epimastigotes and resting stages of *B*. *raabei*, cyst-like amastigotes (CLAs) or "straphangers", were detected in the dock bugs' digestive tract including all segments of the midgut (M1-M4), as well as ileum and rectum, constituting the hindgut (Fig 1). However, no parasites were observed in the hemolymph, salivary glands or Malpighian tubules. The M1-M3 midgut segments contained mainly epimastigotes, but occasionally, there were also single CLAs, not attached to the epimastigotes' flagella. The regular cyst formation was observed in the M4 segment and in the hindgut.

**Fig 2:**
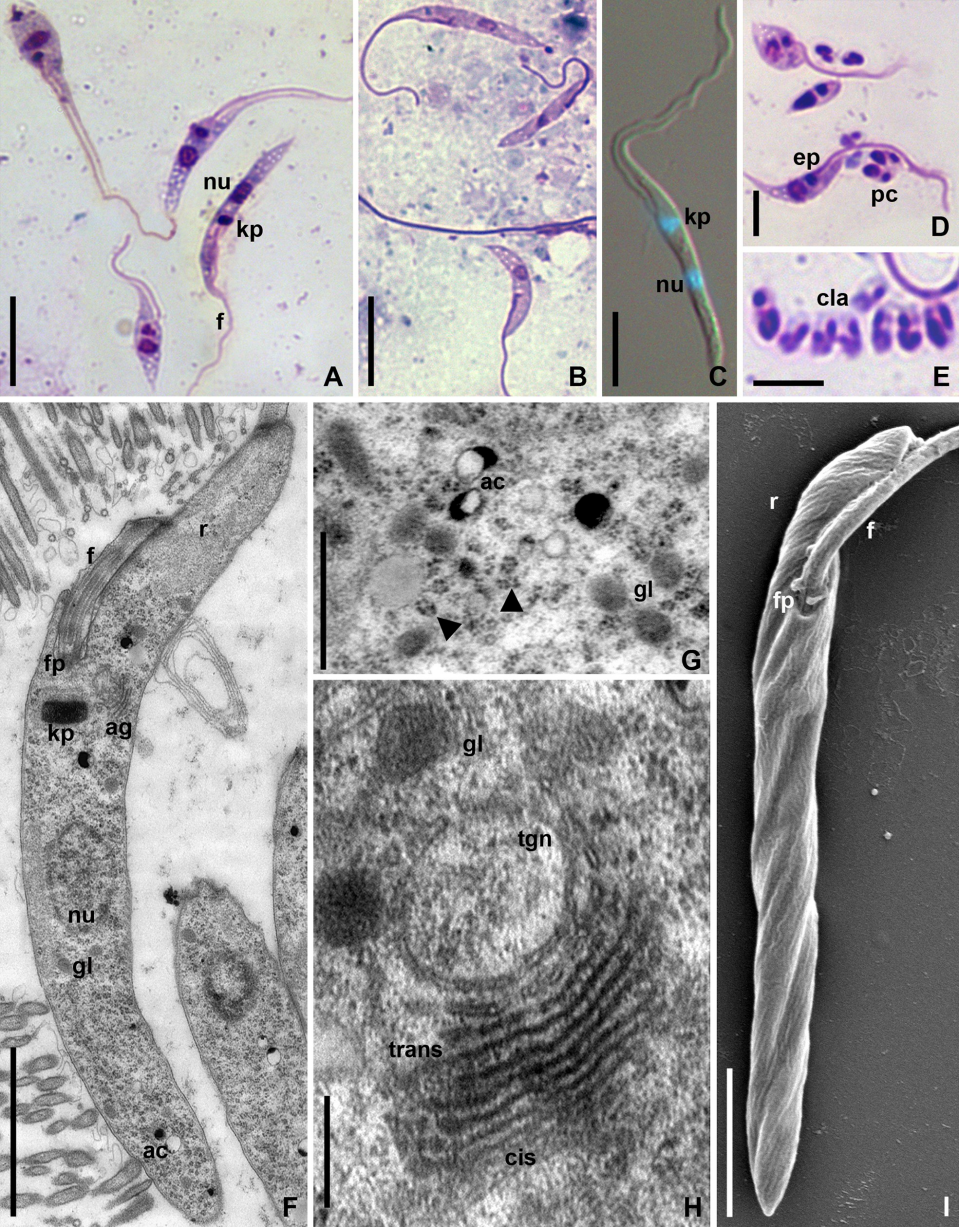
Morphology of *Blastocrithidia raabei* (light microscopy, SEM and TEM). **A.** Epimastigotes from the M3 segment of the host's midgut; **B**. Epimastigotes of "*B*. *r*. *rostrata*" on the archival slide # SP200; **C.** Epimastigote in the culture 123Cor; **D, E.** Formation of CLAs in the rectum; **F, J.** General view of an epimastigote; **G.** Fragment of the cytoplasm showing polysomes (arrowheads); **H.** Golgi complex. **A, B, D, E**–Giemsa staining; **C**–overlaid DIC and DAPI; **F–I–**TEM; **J**–SEM. **ac**–acidocalcisomes; **ag**–Golgi complex; **cis**–*cis* face of Golgi complex; **cla**–cyst-like amastigotes; **ep**–epimastigote; **f**–flagellum; **fp**–flagellar pocket; **gl**–glycosomes; **kp**–kinetoplast; **nu**–nucleus; **pc**–pre-CLA; **r**–rostrum; **tgn**–*trans*-Golgi network; **trans**–*trans* face of Golgi complex. Scale bars: 10 μm (**A, B**); 5 μm (**C, D)** 2 μm (**E**); 2 μm (**F, I**); 0.5 μm (**G**); 0.25 μm (**H**).

The shape and size of epimastigotes varied significantly with almost 2-fold difference in the mean total length values between the cells from the M3 segment and rectum (Fig 2A–2D; Table 1). However, they displayed common traits. The nucleus and kinetoplast were positioned in the middle part of the cell (Table 1; Fig 2A, 2B and 2D). No other DNA-containing structures were detected with DAPI staining (Fig 2C). The anterior part of the cell connected to the flagellum was significantly narrowed and formed a "rostrum"—an elongated structure, typically about one half of the cell length or longer (Fig 2A–2C, 2F and 2G). One more characteristic trait of epimastigotes in this species was the accumulation of clear vacuoles in the posterior end of the cell (Fig 2A and 2B). Epimastigotes on the type slides of *B*. *raabei rostrata* Podlipaev 1988 possessed the same features (Fig 2B) and, taking into account the heterogeneous composition of this material (cells originated from different intestinal parts), did not differ in size from other isolates analyzed here (Table 1).

**Table 1:** Morphometry of *B*. *raabei* cells (N = 25).

	Length	Width	Flagellum	Nucleus	K-A	N-A
**Epimastigotes in the culture**						
123Cor	23,4 ± 3,3	2,5 ± 0,4	28,5 ± 4,9	2,5 ± 0,4	10,8 ± 1,8	14,5 ± 1,9
Novgorod Oblast	(27,5–16,9)	(2,8–1,8)	(34,9–16,0)	(3,1–1,7)	(13,7–6,8)	(17,9–10,6)
**Epimastigotes in the M3 midgut segment**						
123Cor	19,6 ± 3,6	2,0 ± 0,4	32,7 ± 8,0	2,2 ± 0,4	8,4 ± 1,6	11,3 ± 1,9
Novgorod Oblast	(33,2–14,7)	(2,7–1,4)	(56,0–21,4)	(3,2–1,4)	(12,0–5,6)	(16,6–8,2)
174Cor	20,2 ± 3,5	1,8 ± 0,4	26,9 ± 5,7	2,3 ± 0,3	8,1 ± 2,3	11,5 ± 0,6
Leningrad Oblast	(28,1–14,1)	(2,8–1,2)	(37,0–17,0)	(2,8–1,8)	(14,6–4,9)	(16,2–6,6)
85Cor	19,2 ± 2,8	2,1 ± 0,2	32,9 ± 7,2	2,1 ± 0,2	8,7 ± 0,7	11,5 ± 0,5
Pskov Oblast	(26,2–13,8)	(2,7–1,7)	(47,5–13,9)	(2,6–1,8)	(10,0–7,2)	(12,6–10,4)
216Cor	19,9 ± 4,0	2,0 ± 0,3	32,7 ± 7,3	2,1 ± 0,3	8,5 ± 1,1	11,3 ± 1,2
Kurgan Oblast	(28,7–13,4)	(2,7–1,5)	(52,1–19,4)	(2,7–1,3)	(12,0–6,9)	(12,5–9,2)
**Epimastigotes in the rectum**						
123Cor	11,2 ± 2,6	1,9 ± 0,6	18,0 ± 4,1	1,9 ± 0,3	3,5 ± 1,1	5,9 ± 1,3
Novgorod Oblast	(15,0–6,4)	(4,0–1,4)	(24,5–9,4)	(2,5–1,3)	(5,6–1,5)	(8,1–3,6)
**Amastigotes in the rectum**						
123Cor	3,0 ± 0,7	1,8 ± 0,2	N/A	1,4 ± 0,3	N/A	N/A
Novgorod Oblast	(6,0–2,5)	(2,4–1,2)		(2,4–1,0)		
**Epimastigotes on archival total smears**						
546Co	19,2 ± 3,0	2,4 ± 0,3	26,3 ± 4,6	2,1 ± 0,3	9,0 ± 0,7	10,9 ± 0,7
Orenburg Oblast	(26,4–12,9)	(3,1–1,5)	(33,2–19,0)	(3,1–1,9)	(10,2–7,0)	(13,1–9,9)
SP200 (*B*. *raabei rostrata*)	17,7 ± 3,2	2,0 ± 0,3	18,4 ± 5,8	2,0 ± 0,4	7,8 ± 2,4	10,9 ± 2,7
Kazakhstan	(23,9–11,4)	(2,8–1,4)	(31,0–9,8)	(2,8–1,5)	(12,7–3,4)	(15,9–4,9)

N-A is the distance between the nucleus and the anterior end of the cell. K-A is the distance between the kinetoplast and the anterior end of the cell. All the measurements are in μm.

As in most known *Blastocrithidia* spp., the development of CLAs was associated with the flagella of mother epimastigotes (Fig 2D). Usually 1–3 pre-CLAs were attached to the epimastigote flagellum. Free mature CLAs often appeared in the rectal contents in groups of 10 or more (Fig 2E).

#### Electron microscopy

Epimastigotes had typical trypanosomatid nucleus and compact kinetoplast with rod-shaped profile sized 0.66 ± 0.03 × 0.27 ± 0.01 **μ**m (Fig 2F). The cytoplasm of epimastigotes was of medium electron density and very rich in ribosomes, which were organized into polysomes (Fig 2F and 2G). It also contained a moderate number of glycosomes and acidocalcisomes (Fig 2F and 2G). These cells featured a well-developed Golgi complex with numerous cisternae and conspicuous *trans*-Golgi network (Fig 2H). In contrast to other trypanosomatids, in *B*. *raabei* these structures were located laterally in front of the kinetoplast with *trans* face oriented towards the flagellar pocket (Fig 2F). The flagellar pocket opened laterally and the emerging flagellum tightly adhered to the rostrum, but no additional structures ensuring this contact were detected (Fig 2F and 2I).

### Development of *B*. *raabei* in the intestine

**Fig 3:**
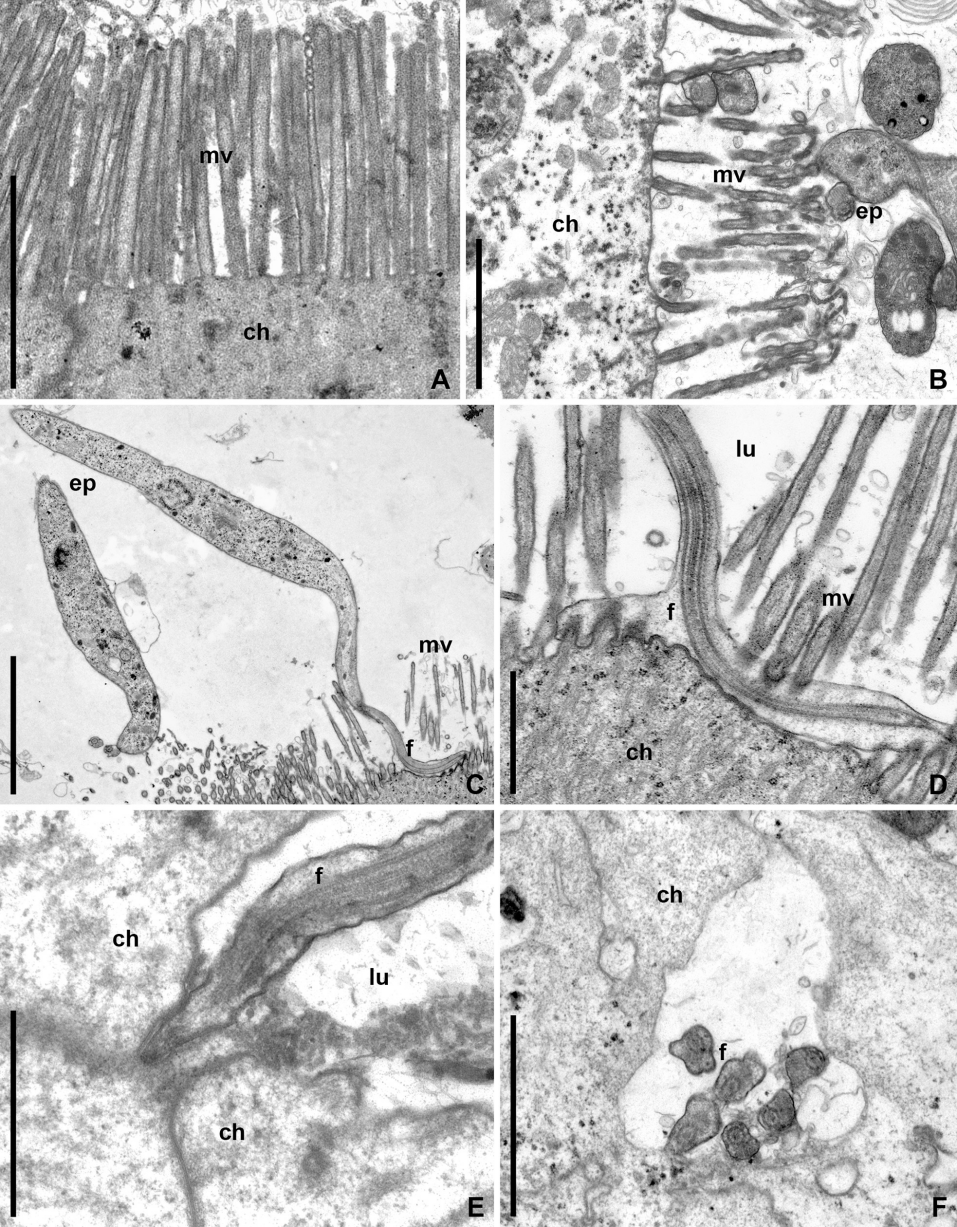
Development of *B*. *raabei* in the M2 and M3 midgut segments (TEM). **A.** Intact brush border of the M2 epithelium; **B.** Brush border degradation in the infected region of M2 in the same dock bug; **C, D.** Attachment of parasites to brush border in M2 and M3 segments of the midgut, respectively; **E, F.** Penetration of epimastigotes' flagella between epithelial cells of M3. **ep**–epimastigote; **f**–flagellum; **ch**–host's epitheliocyte; **lu**–gut lumen; **mv**–microvilli. Scale bars: **A, B, F**– 2 μm; **C**– 5 μm; **D, E**– 1 μm.

In the anterior (M1 –M3) segments of the midgut, the epimastigotes were observed both free in the lumen and attached to the apical surface of the host intestinal epithelium. The intact brush border of host enterocytes was formed by microvilli adjacent to each other and sizing 2–2.5 μm in length and ~ 0.2 μm in diameter (Fig 3A). The flagella of attached epimastigotes penetrated between the microvilli to the apical surface of epitheliocytes, to which they tightly adhered with their lateral surface. The flagellar tips enlarged and seized the adjacent microvilli (Fig 3C and 3D). As a result, brush border degradation was observed in places of accumulation of the attached parasites (Fig 3B–3D).

**Fig 4:**
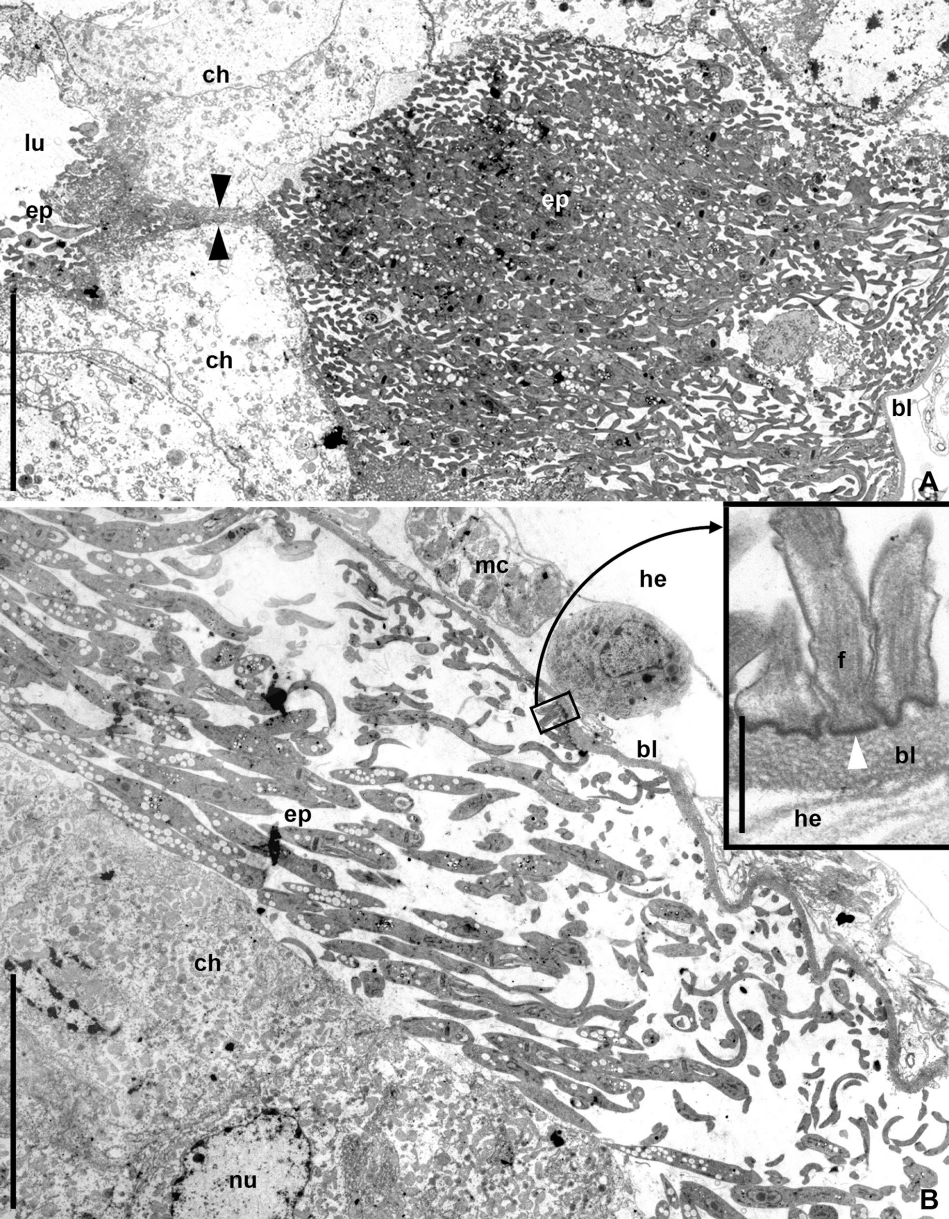
Traversal of *B*. *raabei* through the M3 midgut segment epithelium (TEM). **A.** Epimastigotes passing between host's epithelial cells (arrowheads) from the gut lumen to the space under the basal lamina; **B.** Basal lamina detached from the epithelium by a large number of epimastigotes; **Inset**. Attachment of parasite flagella to basal lamina by hemidesmosomes. **bl**–basal lamina; **ep**–epimastigote; **f**–flagellum; **ch**–host's epitheliocyte; **he**–hemocoel; **lu**–gut lumen; **mc**–muscle cell; **nu**–nucleus of the host cell. Scale bars: **A, B**– 12 μm; **Inset**– 0.8 μm.

In the posterior part of the M3 segment, the path of the free epimastigotes bifurcated: while some of them started attacking the intestinal wall, others continued moving down the digestive tract. The first group passed to the basal part of the midgut epithelium mainly through intercellular spaces (Fig 3E and 3F) and reached the basal lamina, which gradually delaminated, due to extensive congestion of epimastigotes under it (Fig 4A and 4B). That was also accompanied by degradation of epitheliocytes, through the cytoplasm of which individual flagellates started passing. In the cavities formed under the basal lamina, the epimastigotes continued dividing. Some of them attached to the lamina using flagellar tips with the formation of hemidesmosomes (Fig 4B, inset). No disruption of the basal lamina was observed, however individual epimastigotes were occasionally detected between the cells of the coelomic epithelium, covering the intestine from the outside.

**Fig 5:**
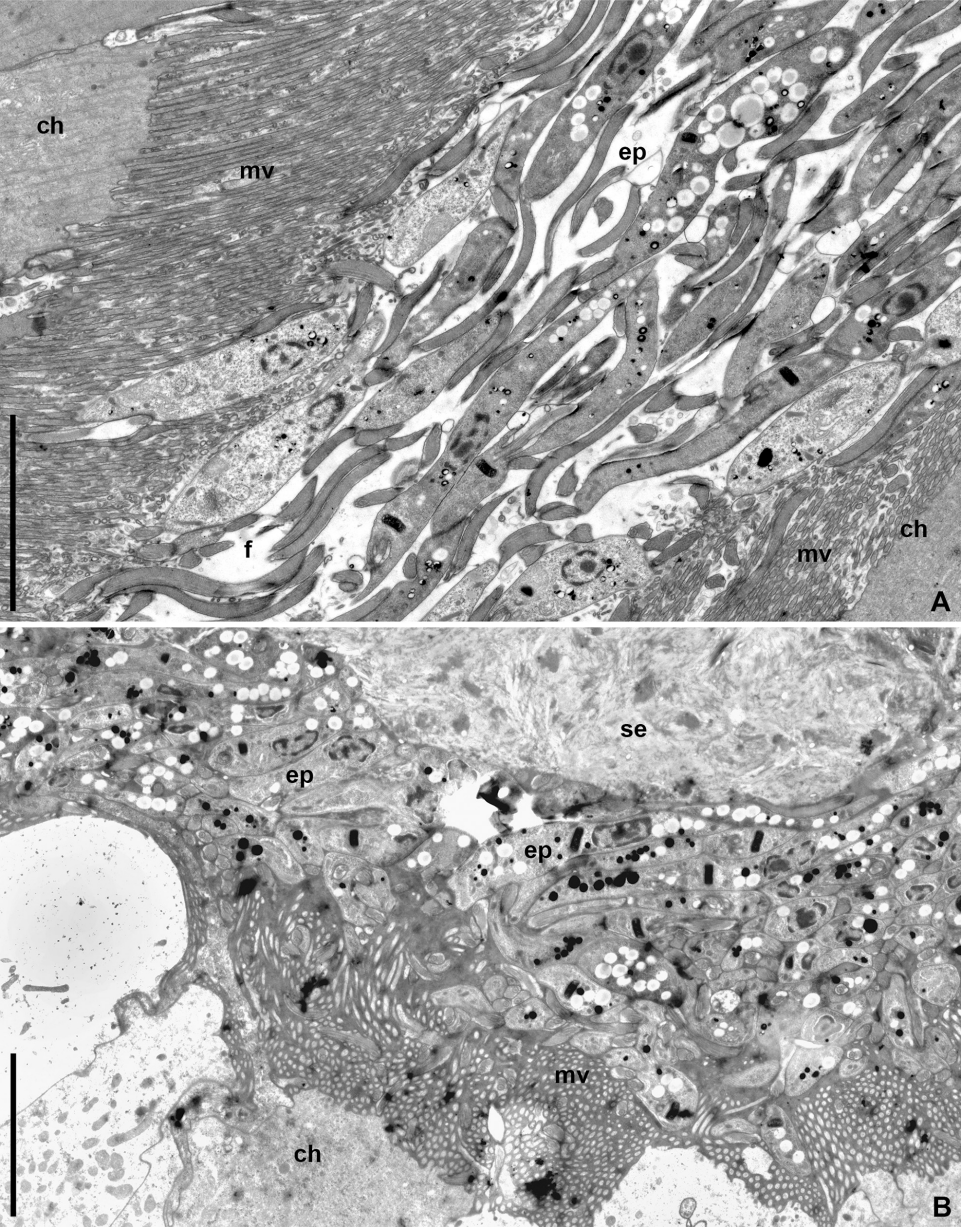
Parasites' penetration into the constricted region and M4B midgut segment (TEM). **A.** Numerous epimastigotes in the constricted region lumen; **B.** Mass of flagellates in the slit-like space between the enterocytes' brush border and the secretory plug in M4B segment. **ch**–host's epitheliocyte; **ep**–epimastigote; **f**–flagellum; **mv**–microvilli; **se**–secretory plug. Scale bars: **A, B**– 10 μm.

Another group of epimastigotes that formed in the posterior part of the M3 segment, accumulated at the entrance to the constricted region (Figs 1B and 5A). The lumen of this part of the midgut was occluded with the hypertrophied (up to 10 μm in length) and tightly adjoining to each other microvilli of host enterocytes (Fig 5A). The flagellates actively overcame this barrier and entered the M4B segment (Fig 1A and 1B). The glandular cells of this intestinal part produced a viscous secret forming a plug impassable for the parasites. Therefore, all flagellates getting into M4B were displaced to its periphery and extremely compressed between the secret and enterocyte microvilli (Fig 5B).

**Fig 6:**
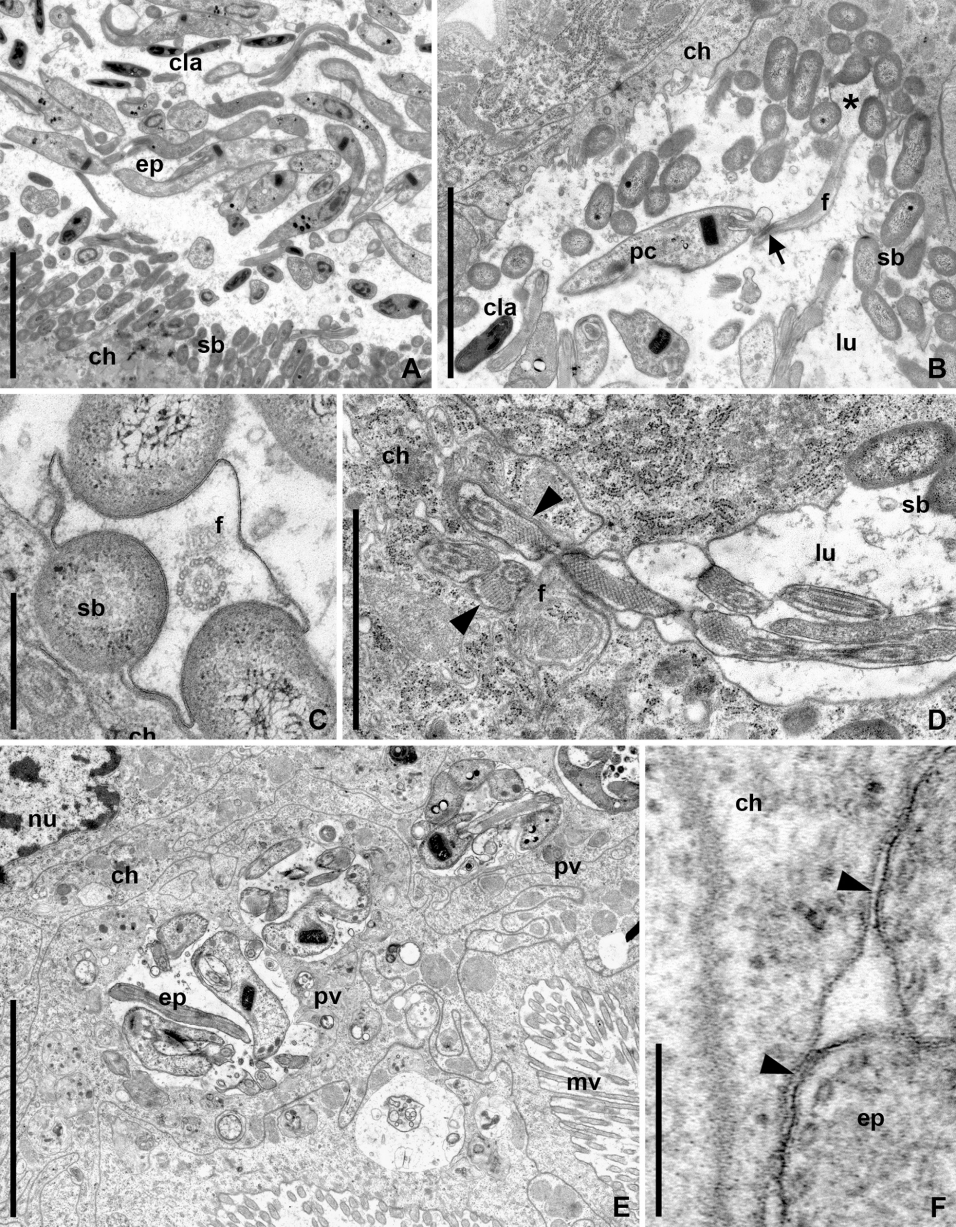
Development of *B*. *raabei* in the M4 midgut segment (TEM). **A.** Epimastigotes and CLAs of *B*. *raabei* in the crypt of M4 segment; **B.** Pre-CLA on the flagellum of mother epimastigote anchored between bacterial cells; **C.** Epimastigote flagellum grabbing the cells of symbiotic bacteria; **D.** Parasitophorous vacuole formation: invagination of the host cell plasmalemma (arrowheads) by parasites' flagella; **E.** Parasitophorous vacuoles with epimastigotes; **F.** Membrane of the parasitophorous vacuole. **ch**–host's epitheliocyte; **ep**–epimastigote; **f**–flagellum; **lu**–gut lumen; **mv**–microvilli; **nu**–nucleus of the host cell; **pc**—pre-CLAs; **pv**–parasitophorous vacuole; **sb**–symbiotic bacteria. Asterisk–anchored dilated flagellar tip; arrow–contact between flagella of epimastigote and early stage of CLA development; arrowheads–host cell / parasitophorous vacuole plasmalemma; Scale bars: **A, D**– 2 μm; **B**– 4 μm; **C**– 0.5 μm; **E**– 5 μm; **F**– 0.2 μm.

The M4 midgut segment (Fig 1) consisted of numerous sacciform crypts opening into the central canal. The surface of the crypts was covered by the cells of symbiotic bacteria (Fig 6A and 6B). These bacteria made the plasmalemma of enterocytes to invaginate and partially plunged into the cytoplasm of the latter (Fig 6B and 6C). The cells of *B*. *raabei* were observed in multitude within the lumina of the crypts (Fig 6A). Many epimastigotes were attached to the crypts' surface via modified flagella using the bacterial cells as a substrate (Fig 6B and 6C). The attached parasites started cyst formation with the daughter cells formed on the mother epimastigote's flagellum. Various developmental stages of this process were observed in the M4 segment (Fig 6A and 6B). Some epimastigotes used their flagella to invoke invagination of the host cell plasmalemma (Fig 6D). This eventually led to the formation of the parasitophorous vacuoles, surrounded by host membrane and containing one or more flagellates (Fig 6E and 6F).

**Fig 7:**
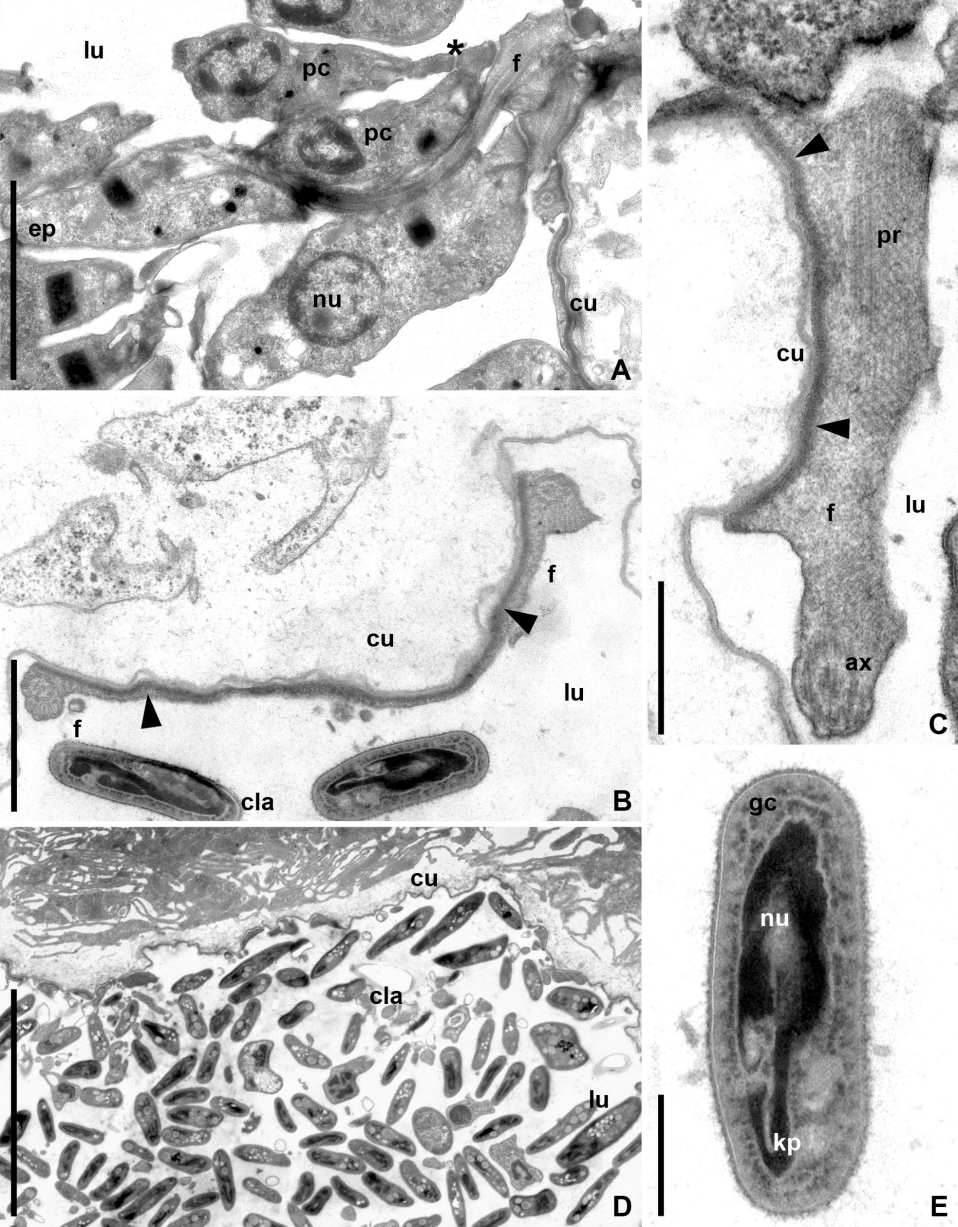
Development of *B*. *raabei* in the rectum (TEM). **A.** Pre-CLAs attached to each other and to mother epimastigote's flagellum; **B, C.** Attachment of epimastigotes to the rectal cuticle by lateral flagellar surface with the use of hemidesmosomes; **D.** Accumulation of CLAs in the rectum; **E.** Mature CLA structure. **ax**–axoneme; **cu**–cuticle; **ep**–epimastigote; **f**–flagellum; **gc**–granular cytoplasm layer; **kp–**kinetoplast; **lu**–gut lumen; **nu**–nucleus; **pc**—pre-CLAs; **pr**–paraxial rod. Asterisk–flagellar contact between pre-CLAs; arrowheads–hemidesmosomes. Scale bars: **A**– 2 μm; **B**– 1 μm; **C**– 0.5 μm; **D**– 6 μm; **E**– 0.8 μm.

From the M4 segment lumen the parasites advanced to the hindgut. In the rectum, most epimastigotes were localized on the surface of the cuticular lining (Fig 7A). They attached to the epicuticle with the lateral flagellar surface using hemidesmosomes (Fig 7B and 7C). Free epimastigotes often formed rosettes. An intensive cyst formation was observed in the rectum with over 70% of epimastigotes (both free and attached) involved into this process (Fig 7B). Mature CLAs displayed all typical traits of such cells: highly compacted nucleus and kinetoplast, a dense layer of fine granular cytoplasm under plasmalemma and no traces of flagellum (Fig 7E).

### Molecular phylogenetic analyses

The diversity of monoxenous trypanosomatids has been previously studied using mainly two different molecular markers– 18S rRNA and SL RNA [5], for which the datasets overlap only partially. Therefore, we decided to use both of them in order to better understand the phylogenetic affinities of *B*. *raabei*.

**Fig 8:**
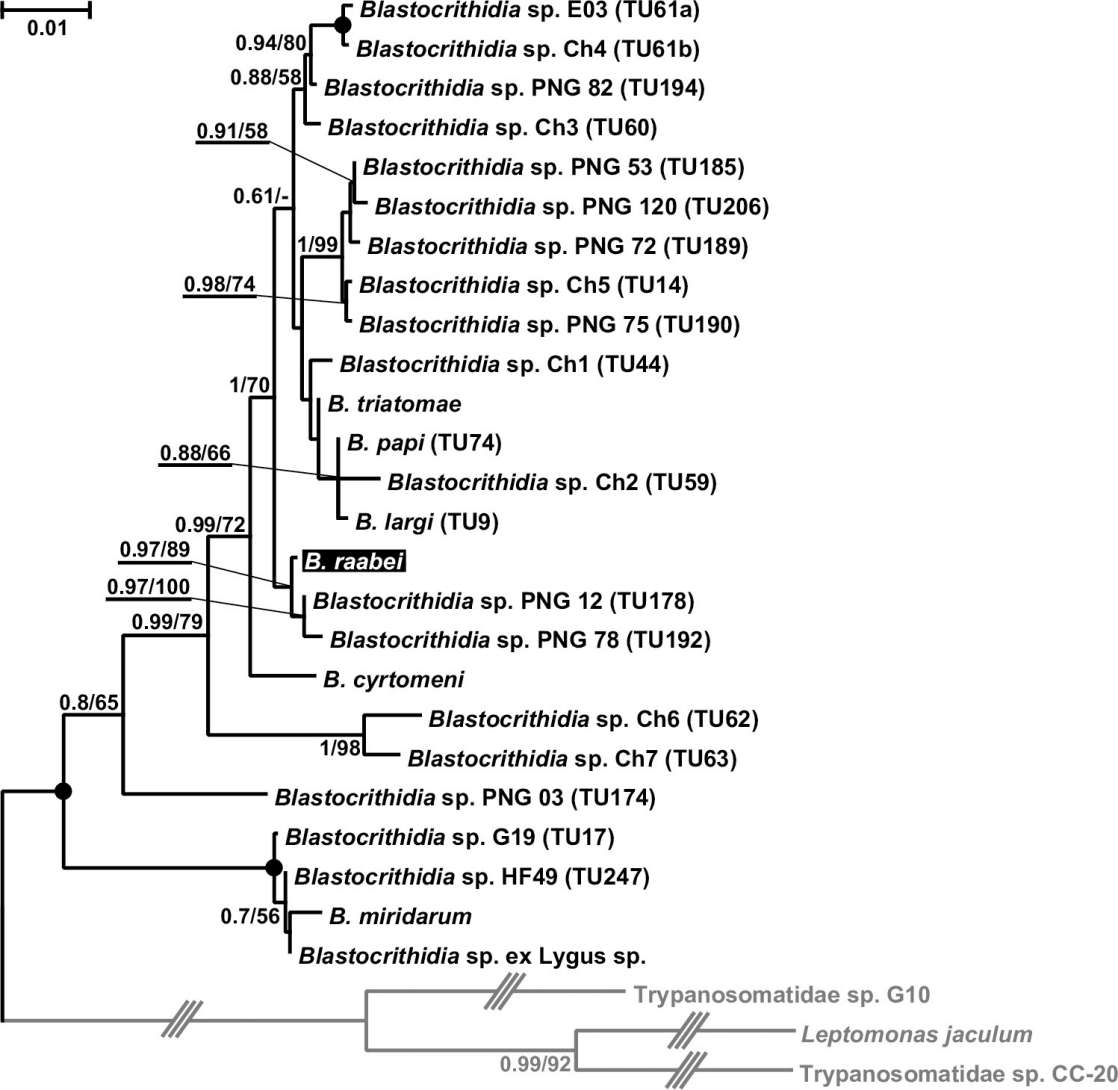
Maximum likelihood phylogenetic tree based on 18S ribosomal RNA gene sequences. Numbers at nodes indicate posterior probability and bootstrap percentage, respectively. Values less than 0.5 and 50% are replaced with dashes or omitted. Nodes having 1.0 posterior probability, 100% bootstrap support are marked with black circles. Triple-crossed branches are at 1/3 of their original lengths. The tree is rooted with the sequences of three cyst-forming trypanosomatids of the *Leptomonas jaculum* lineage. The scale bar denotes number of substitutions per site. The species under study (*Blastocrithidia raabei*) is highlighted.

On the 18S rRNA gene-based tree (Fig 8), the closest relatives of *B*. *raabei* were *TU*178 and *TU*192 represented by the trypanosomatid isolates PNG12 from *Kanigara fumosa* (Rhyparochromidae) and PNG78 from *Leptocorisa acuta* (Alydidae), respectively, both isolated in Papua New Guinea [56]. The sequences of these two TUs differed from that of *B*. *raabei* by 4 substitutions and 1 indel (PNG12) and 8 substitutions and 1 indel (PNG78). Although there is no universally accepted similarity threshold for this molecular marker in trypanosomatids, this variation is certainly interspecific, since the two undoubtedly separate species (*Blastocrithidia papi* and *B*. *largi*) differ by only two substitutions in the whole sequences of this gene [29]. Meanwhile, the 18S rRNA gene sequences of *B*. *raabei* demonstrated variation in one nucleotide position, where C, T or a missing base were detected. This was observed not only in dock bug populations, but also in the sample 546Co from Orenburg Oblast.

**Fig 9:**
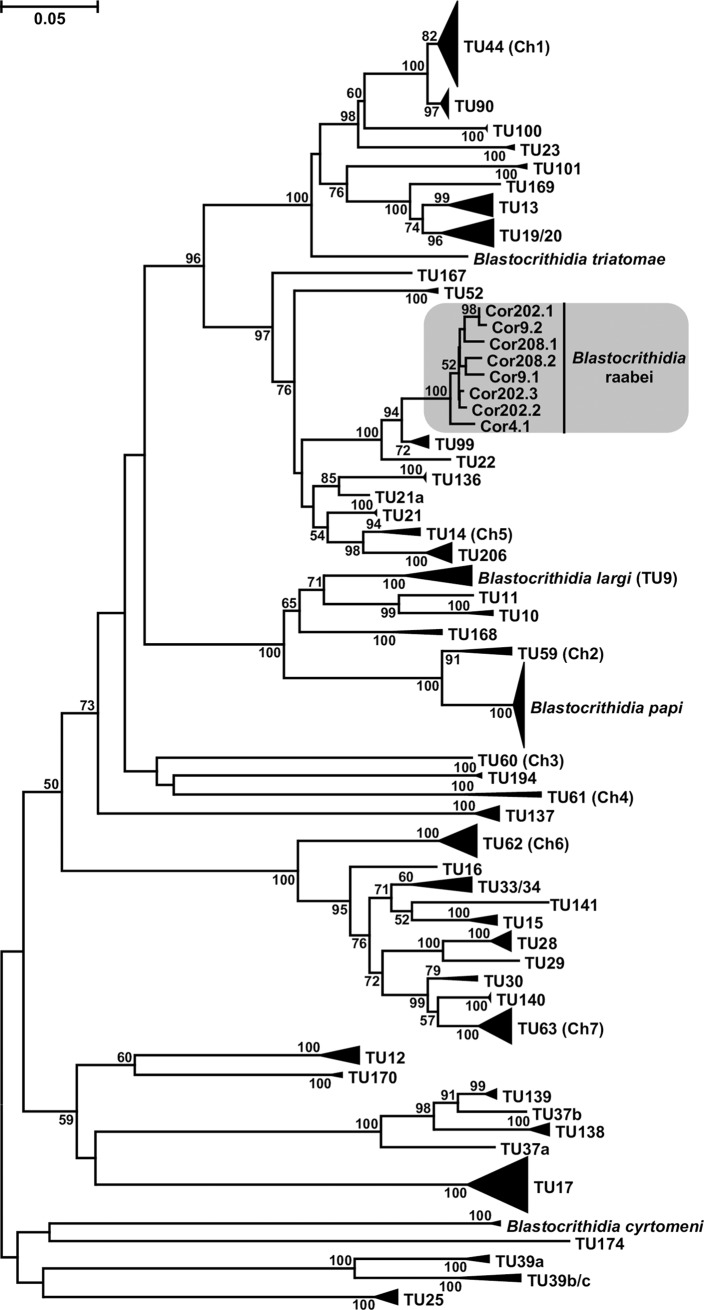
SL RNA / 5S rRNA gene-based neighbor-joining dendrogram of *Blastocrithidia* spp. The dendrogram is rooted at the midpoint. All clades corresponding to typing units, except that of *B*. *raabei* (highlighted) are collapsed. Numbers at nodes indicate bootstrap percentage, values less than 50% are not shown. The scale bar corresponds to number of substitutions per site.

The analysis based on the SL RNA / 5S rRNA gene region showed another relative of *B*. *raabei*, which may be even closer to it than the two abovementioned TUs (Fig 9). This was TU99 represented by the trypanosomatid isolate 232VB (from *Repipta* sp., Reduviidae), the sequences of which had identity to those of the species under study in the range of 86.2–89.9%, i.e. approaching the proposed specific threshold of 90% [45]. The variation of this marker sequence in *B*. *raabei* samples ranged between 93.5 and 99.7%, confirming that this is a single species.

In agreement with the previously published data on the bacterial symbionts of Coreoidea [38, 57], the 16S rRNA gene sequences from the M4 midgut segment of the dock bugs, obtained in this work, showed over 99% identity to those of *Burkholderia* sp. from *C*. *marginatus* and *Dicranocephalus agilis* (Stenocephalidae).

## Discussion

### Presence in the host's hemolymph

Some dixenous trypanosomatids pass from the gut to the hemolymph of the insect host in order to reach the salivary glands (which have no direct connection with the digestive tract) and, thereby, ensure their transmission to a plant or a vertebrate [26]. In monoxenous species, the hemolymph infection is very rare, since it does not provide a considerable advantage to the parasites, but requires elaboration of expensive mechanisms for exit from the gut and defense against immune system of the host. However, in *Herpetomonas swainei*, parasite of the jack pine sawfly *Neodiprion swainei*, stages in the hemolymph guarantee transmission between the host developmental phases [58]. In *Leptomonas pyrrhocoris* from the firebug *Pyrrhocoris apterus*, hemolymph infection increases efficiency of horizontal transmission owing to frequent cannibalism in the host populations [59].

*Blastocrithidia raabei*, along with two other species of this genus, *B*. *caliroae* from the pear slug *Caliroa cerasi* and *B*. *cyrtomeni* from the burrowing bug *Cyrtomenus bergi*, have been documented in the host hemolymph [35, 60, 61]. However, all these cases need further independent confirmation. Firstly, examination with light microscope did not allow observing the image of parasites' traversal through the gut wall. Secondly, the method of hemolymph sampling was not specified, therefore contamination with gut contents cannot be excluded. Thirdly, mixed infections were quite likely in all three cases, since in addition to epimastigotes, the diagnoses and/or accompanying illustrations contained morphotypes not typical for the genus *Blastocrithidia*: promastigotes [35, 60, 61] and opisthomastigotes [61]. As we proposed previously, the promastigotes, observed by Lipa, might belong to *Phytomonas lipae*, a recently described dixenous parasite, whose development includes obligatory stages in the hemolymph of *Coreus marginatus* [55].

Our analysis of the hemolymph from 53 dock bugs infected with *B*. *raabei* did not reveal any parasite cells, thus contradicting Lipa's observation [35]. This discordance may be a result of different methods of hemolymph sampling: independent from gut isolation (in our study) and, supposedly, combined with it (in Lipa's case). This assumption is further supported by the fact that Lipa's preparations of hemolymph contained CLAs and rosettes of epimastigotes [35]. According to our data, the former are abundant and the latter are exclusively present in the rectum, the terminal part of the intestine, the contents of which could be discharged upon isolation. Thus, we argue that the development of *B*. *raabei* is restricted to the host gut.

### Peculiar development in the gut

In *C*. *marginatus*, like in other phytophagous bugs of the superfamilies Lygaeoidea and Coreoidea, the digestive tract has a very unusual organization. Its anterior (foregut and M1-M3 midgut segments) and posterior (symbiont-containing M4 midgut segment and hindgut) parts are effectively isolated from each other by two intermediate segments (constricted region and M4B), representing a barrier impassable for food fluid and microorganisms [38, 62]. This poses a significant challenge for the flagellates venturing to parasitize such insects. They must find a way to reach a location, wherefrom their infective forms can be discharged and, thereby, used for transmission. *Phytomonas* spp., inhabiting such bugs, head for the salivary glands, but *Blastocrithidia* is not adapted to parasitism in plants and, therefore, must follow the ancestral path for the monoxenous trypanosomatids, i.e. advance along the digestive tract and arrive to the rectum.

In the anterior segments of the midgut (M1 –M3), the interaction of *B*. *raabei* epimastigotes with the host (attachment with the dilated flagellar tip in the zone of microvilli causing their degradation) is similar to that in other species of the genus [33,63–65]. However, due to the peculiarity of the host midgut morphology and physiology, starting from the posterior funnel-shaped portion of the M3 segment, the behavior of *B*. *raabei* becomes strikingly different from what has been described in other species. A large mass of flagellates accumulate there in front of the "bottleneck", represented by the constricted region featuring an extremely narrow canal bordered with the densely packed elongated microvilli and followed by the M4B producing a viscous secret, completely filling the lumen. The symbiotic bacteria of the genus *Burkholderia* use intensive flagellar movement to overcome these barriers. However, the motility alone appears to be insufficient, as judged by the inability of other flagellated bacteria to pass through the filtering regions. Therefore, a putative secretolytic activity was proposed to be the second competence factor for the symbionts [38]. In *Blastocrithidia raabei*, which successfully reaches the symbiontophorous M4 segment, there are two features, which distinguish this species from other studied members of the genus. These are a hypertrophied rostrum and a well-developed Golgi complex. The first trait ensures undulation of the larger part of the cell body, increasing efficiency of the movement in the tight space of the filtering segments [66, 67]. The second one is likely responsible for the secretolytic activity, which is facilitated by a convenient location of the Golgi complex near the base of the flagellar pocket, the main gate of the trypanosomatid cell for the exchange with the environment [68].

The fate of another group of *B*. *raabei* epimastigotes in the posterior portion of the M3 segment is even more peculiar. They migrate through the intercellular spaces in the intestinal epithelium, exhibiting the typical aggressive behavior of *Phytomonas* spp. and some trypanosomes [39, 69–72]. However, while the abovementioned dixenous parasites head for the hemolymph and then to the salivary glands, wherefrom the infective stages can be transmitted to a plant or a mammal host, *B*. *raabei* does not cross the basal lamina underlying enterocytes. This appears to be a dead end, since the flagellates become entrapped in a location, which prevents transmission to a new host. It would be reasonable for epimastigotes to use this route as a bypass allowing them to enter the coveted M4 segment from the outer side of the epithelial layer, but this was never observed. We hypothesize that the massive migration to the basal lamina serves a very unusual purpose–suppression of the proper functioning of the downstream segments' epithelium. Indeed, the progressive delamination of the basal lamina led to epitheliocytes' degradation, which was especially pronounced in the cases of multiple migration foci. This may represent a distant analogy to the destruction of the sandfly stomodeal valve by haptomonads, a specialized group of flagellates facilitating the discharge of infective metacyclic promastigotes in *Leishmania* spp. [73].

In the M4 midgut segment, the symbiotic burkholderiae are located in the invaginations of the epitheliocytes' plasmalemma. This ensures retaining of the bacteria on the host cells' surface and increases the area of the contact between them, thus enhancing the metabolic exchange. The attachment of *B*. *raabei* epimastigotes to bacteria is likely a consequence of the deficit of the free epitheliocytes' surface. Yet, some parasites find gaps and penetrate into the host cells in parasitophorous vacuoles. Intracellular stages are typical for many dixenous trypanosomatids and perform various functions [37]. Among monoxenous trypanosomatids this phenomenon has been previously documented only in *Crithidia flexonema* from the water strider *Gerris odontogaster*, where a single cell was located to the parasitophorous vacuole in a Malpighian tubule epitheliocyte [74]. In contrast to this, *B*. *raabei* massively attacks the enterocytes, which is reminiscent of the behavior of some dixenous trypanosomatids in the salivary glands (not the gut!) of the insect host [39, 69–71, 75]. However, in those dixenous species, the intracellular flagellates represent the migrating stages, whereas in the species under study they are not. The reason of this phenomenon in *B*. *raabei* may be the non-canonical function of the M4 midgut segment. Indeed, instead of digestion and absorption, which are the inherent functions of the midgut, this part of the intestine is free of the food flow. Therefore, the nutrients in the lumen should be extremely scarce and, as proposed above, the symbiotic bacteria must be fed through the direct membrane contacts with the host cells. Importantly, while there were many examples of the epitheliocytes' invasion, we never observed the exit of the intracellular flagellates. We posit that the cells of *B*. *raabei* in host's epitheliocytes represent persisting forms, which ensure preservation of infection during host hibernation. As for the extracellular epimastigotes, they form CLAs, likely at the expense of the storage products from the clear vacuoles in the posterior end. Similarly, *Trypanosoma cruzi* utilizes the contents of reservosomes during metacyclogenesis [76].

The behavior of *B*. *raabei* in the hindgut is typical for *Blastocrithidia* spp.: the ileum is passed without entering the Malpighian tubules and the rectal lining is used for the attachment of epimastigotes. The morphology of CLAs and the process of their formation also do not differ from those in other species of the genus [60, 77–80]. Peculiarly, for the attachment in the rectum, *B*. *raabei* uses the extended lateral flagellar surface, rather than a dilated tip or a flattened attachment pad, which are inherent to other trypanosomatids inhabiting this intestinal region [63, 65, 81–86]. However, this mechanism has been previously documented in epimastigotes of African trypanosomes developing in salivary glands or in vitro [87–89]. As in the case of the upstream M4 midgut segment, the rectum of the dock bugs does not contain any food and, therefore, epimastigotes can rely only on their own supplies, supposedly stored in the posterior vacuoles. Thus, it appears that cyst formation in *B*. *raabei* is triggered by two factors: flagellar attachment and deficit of nutrients in the environment, therefore it does not happen in the midgut upstream of the constricted region. Of note, this process occasionally occurs in the epimastigotes attached to the basal lamina, in the location where the nutrients are depleted, especially given the big mass of parasites, accumulating there.

### Taxonomic section

**Class** Kinetoplastea (Honigberg, 1963) Vickerman, 1976

**Subclass** Metakinetoplastina Vickerman, 2004

**Order** Trypanosomatida (Kent, 1880) Hollande, 1952

**Family** Trypanosomatidae (Doflein, 1901) Grobben, 1905

**Genus** *Blastocrithidia* Laird, 1959

***Blastocrithidia raabei*** Lipa, 1966

**Synonymy**: *B*. *raabei rostrata* Podlipaev, 1988

**Morphology**: epimastigotes 6–28 μm long, with a long rostrum constituting in average one half of the total cell length, nucleus and kinetoplast in the middle part of the cell, flagellum length exceeds that of the cell body, numerous clear vacuoles in the posterior part of the cell, Golgi complex at the base of the rostrum, cyst-like amastigotes measure 3.0 × 1.8 μm.

**Type host**: *Coreus marginatus* Linnaeus, 1758 (Heteroptera: Coreidae).

**Location within host**: midgut and rectum (lumen and surface), space between basal lamina and epithelium of M3 midgut segment, parasitophorous vacuoles in the epitheliocytes of the M4 segment.

**Distribution**: Eurasia; the area, apparently coincides with that of the host.

**Type material**: hapantotype Cor123.1 –Cor123.5 (Giemsa-stained dry smears) deposited in the research collection of the laboratory of Protozoology of the Zoological Institute of the Russian Academy of Sciences along with the axenic culture Cor123.

**Gene sequences**: the species can be identified by the sequences of 18S rRNA, and 5S / SL RNA repeat region (GenBank accession numbers: MN366346-MN366353, MN380289-MN380296, respectively).

**Comments**: The original description by Lipa was based on a mixed infection. The subspecies *B*. *raabei rostrata* Podlipaev, 1988 was proposed to have a distinctive feature–the conspicuous rostrum in epimastigotes. That was a misconception, since although not mentioned in the text, this trait was also present on Lipa's figures. Morphology of the epimastigotes studied by Lipa, Podlipaev and in our work is similar. The slight differences in the average cell length are non-significant in view of this measurement variation within one host individual (Table 1). Our analysis of molecular sequences suggests that distant dock bug populations in Europe and Asia host the same species of the genus *Blastocrithidia*.

## Conclusions

*Blastocrithidia raabei* does not develop in the hemolymph, but anyway exhibits an aggressive behavior typical for some dixenous parasites. This is a consequence of the non-canonical organization of the gut of its host, the dock bug *Coreus marginatus*, with two barrier segments of the midgut, making this insect refractory to microbial infections. For the successful development in such a host, *Blastocrithidia raabei* employs several distinct functional groups of epimastigotes. In the pre-barrier midgut segments, where the conditions are favorable, the parasites settle, propagate and accumulate resources. On the border with the constricted region, the main group ("strike force") of epimastigotes facing the barrier "go berserk" and fiercely break through the host defenses. Their successful advancement is ensured by two special cellular features, such as the enlarged rostrum and the well-developed Golgi complex. At the same time, the second group of epimastigotes, the putative "sabotage unit", aims to weaken the host's defense. On the other side of the barrier, epimastigotes find themselves in a zone depleted of food sources, a "scorched earth", where they must consume reserved supplies and complete the mission. The role of the cells, passing to the "rear area", i. e. inside the enterocytes of the M4 segment, is not completely understood, but most probably they represent a reserve group acting at a specific moment of parasite's developmental cycle. In sum, *B*. *raabei* is an insistent parasite, which does not retreat in face of difficulties, but uses a complex strategy and functional cell improvements in order to achieve its goal.
